# A large-scale sentiment analysis of tweets pertaining to the 2020 US presidential election

**DOI:** 10.1186/s40537-022-00633-z

**Published:** 2022-06-16

**Authors:** Rao Hamza Ali, Gabriela Pinto, Evelyn Lawrie, Erik J. Linstead

**Affiliations:** Fowler School of Engineering, Chapman University, One University Drive, Orange, California USA

**Keywords:** Sentiment analysis, Natural Language Processing, Twitter analysis, US Elections 2020

## Abstract

We capture the public sentiment towards candidates in the 2020 US Presidential Elections, by analyzing 7.6 million tweets sent out between October 31st and November 9th, 2020. We apply a novel approach to first identify tweets and user accounts in our database that were later deleted or suspended from Twitter. This approach allows us to observe the sentiment held for each presidential candidate across various groups of users and tweets: accessible tweets and accounts, deleted tweets and accounts, and suspended or inaccessible tweets and accounts. We compare the sentiment scores calculated for these groups and provide key insights into the differences. Most notably, we show that deleted tweets, posted after the Election Day, were more favorable to Joe Biden, and the ones posted leading to the Election Day, were more positive about Donald Trump. Also, the older a Twitter account was, the more positive tweets it would post about Joe Biden. The aim of this study is to highlight the importance of conducting sentiment analysis on all posts captured in real time, including those that are now inaccessible, in determining the true sentiments of the opinions around the time of an event.

## Introduction

Twitter is a mature and popular social media platform based on short text posts, to share quick opinions or ideas [1, 2]. Instantaneous communication on Twitter can create a hub of controversy and misinformation on occasions, especially surrounding political issues. About 60 percent of users discuss political issues on sites like Twitter, making social media a popular place that people rely on for political updates [3]. In 2020, Americans cast their vote for the next president of the United States, either for the Republican incumbent, President Donald J. Trump, or the Democratic challenger, former Vice President Joseph R. Biden. As the Election Day of November 3rd loomed closer, more Americans took to Twitter and other social media platforms to voice their political opinions and engage in conversation surrounding the elections. Due to the COVID-19 pandemic, a record number of mail-in ballots were requested, significantly increasing the time it would take to announce the projected winner with confidence [4]. While early results trickled in on Election Day, news outlets could not call the election for four more days, causing an uptick in election-related traffic on Twitter for an extended period of time.

Sentiment Analysis, an area of natural language processing, is the study of measuring a sentiment (positive, negative, or neutral) of a piece of text by analyzing the words used in it [5] and is often used to understand the favorability towards a product, a figure, or an event [6–8]. In this study, we apply sentiment analysis to understand the favorability of each presidential candidate and how it changed as the events surrounding the elections unfolded. This technique has been previously applied to tweets to determine the political temperature on Twitter [6, 9, 10]. In this paper, we focused on the heightened emotional nature of the elections. With both candidates at ends on topics like social justice, climate change, and COVID-19 preventative measures, Americans fiercely shielded their preferred candidate and attacked the other on Twitter. Some users voiced opinions that they would later regret or were deemed incendiary by Twitter, leading to their account suspension or deletion. It becomes imperative to conduct sentiment analysis by viewing the sentiment across these different groups of users, as we hope to explain the volatile favorability of candidates on Twitter.

Application of sentiment analysis on tweets that are only currently available today or on tweets of users who still have an active account, will not measure the actual sentiment observed during the time of the elections. Suppose a user posts multiple incendiary tweets against a candidate, leading to a flurry of positive posts in response from supporters of that candidate. If the user then removes those tweets and we measure the sentiment on only active tweets, then it will come across as that candidate having a completely positive sentiment, across all users. But in reality there were posts that held unfavorable sentiment towards that candidate and the analysis should account for that. Twitter’s official API only gives access to active or available tweets and accounts [11], so any sentiment analysis performed on tweets that were not recorded as soon as they were posted, and instead uses tweets using the API at a later date, will not compute the raw sentiments right in the middle of the event. Our study takes this notion into account and highlights the different sentiments observed across active, deleted, suspended, and unauthorized groups to showcase the raw sentiments of users during the election.

Inclusion of deleted tweets in this study will further allow information to be gleaned that encompasses perspectives that Twitter users felt the need to retract. This ties into the prevalence of misinformation on social media platforms like Twitter [12, 13]. Since the beginning of the COVID-19 pandemic, global mental health issues have increased, often fueled by repeated pandemic-related misinformation being spread on social media [14]. Understanding this spread, which is often followed by a flurry of retractions and suspensions, is another motivation of this study. Observing how users behave when it is seemingly easy to retract the information from a public platform at a later date, can help identify ways to combat misinformation on such platforms.

Our analysis spans ten days, starting from October 31st until November 9th, 2020, over which we actively stored 7.6 million tweets that mentioned either candidate. We then cross-referenced Twitter’s API to check which users or tweets had a changed status (suspended, deleted, unauthorized). After applying sentiment analysis on each tweet, we can then ascertain the average sentiment for each candidate and further express it per different user and tweet status types. We hope to understand the construct of the sentiment surrounding the presidential candidates and how different status types fuel the overall sentiment on Twitter during a monumental political event.

In the following sections, we will first provide a brief overview of similar studies and how our work adds a new dimension to the area of research. Next, we will introduce the materials and methods used for the study, followed by a detailed explanation of the results. Lastly, we will discuss the implications of the results and will state our conclusion.

## Related work

Studies have previously used Twitter to study communication and discussions during the U.S. Presidential elections [15–19]. Yaqub et al. [20] performed sentiment analysis on Twitter data collected for ten days before and after the Election Day . Their objective was to associate the sentiment of the discussions with features of Twitter users, such as their number of followers, duration of activity, and number of tweets. Joyce and Deng [21] focused on the relationship between the tweet’s sentiment and the hashtag topic associated with the tweet. In our research, we too conduct the analysis on tweets collected both before and after the Election Day. However, we correlate the sentiment analysis of the tweets to the status of the tweet and the status of its author. In doing so, we are able to find groups of users who behave differently in a volatile election cycle.

Almuhimedi et al. [22] first presented the approach to quantify and assess deleted tweets on the platform, and Zhou et al. [23] further modeled the deletion behavior and applied sentiment analysis on the deleted tweets. Meeks [24] used the approach to examine deleted tweets from politicians to show how political campaigns strategically hide and present certain information to voters. Our study is a novel addition to this area of research, as we use the deleted tweets to provide a commentary on the overall sentiment observed on Twitter for a major political event. Exclusion of deleted or inaccessible tweets from sentiment analysis would result in an incomplete picture regarding the opinions prevalent around the election cycle.

## Materials and methods

### Data collection and cleaning

We used the tweepy package [25] in Python programming language [26], in connection with a Twitter developer API [27], to save a stream of tweets that mentioned the keywords ‘Trump, Donald Trump, trump, Biden, Joe Biden, biden’, posted between October 31st, 2020 and November 9th, 2020. The stream option allows us to capture, in real-time, any tweet that fits the criteria and store it in a file. This way, even if a tweet is deleted later from Twitter, we still have access to the tweet’s text. We collected 12 million tweets in total spanning more than a dozen different languages, recording their creation date and time (in GMT), the creator’s account handle, the text’s language, and the tweet’s unique identifier. We did not collect retweets, which occur when an account tweets another account’s tweet without adding additional text, as we wanted to look at original text explicitly. We initially relied on the language label that Twitter gave the tweets as the language of the text. The API documentation mentions that the language code assigned to each tweet is “best-effort” [28] and can be inaccurate, which we found to be true after a manual inspection of a subset of tweets collected. Instead, we used the polyglot [29] package in Python to assign the correct language code. The package uses Google’s Compact Language Detector [30] as a backend which is a neural network model for language identification. Each tweet’s text is analyzed by the model and a language code is assigned to it. This approach corrected a significant number of mislabels we had observed earlier.

**Fig. 1:**
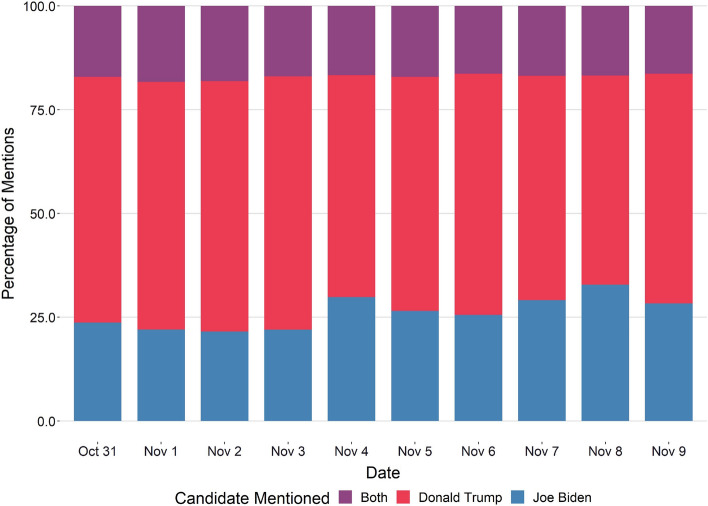
Percentage share of a presidential candidate mentioned in a tweet per day

Although most tweets were in English, roughly a million tweets collected were in Spanish, Portuguese, and French. While sentiment analysis of text in English is a widely studied topic: popular natural language processing toolkits, including NLTK [31], TextBlob [32], CoreNLP [33], and spaCy [34], all provide functionality for English text sentiment analysis; researchers are yet to replicate the success of the analysis to other languages. Because there is a lack of a universal lexicon for other languages that can be used to train the model, sentiment analysis of non-English text, specifically short texts like tweets, requires extensive preprocessing and is not as accurate as the English counterpart [6, 35]. Brooke et al. studied cross-language sentiment analysis from English to Spanish and found that traditional mechanisms and inclusion of translations resulted in a worse off performance for Spanish texts compared to English documents [36]. We will need to create a separate model for each language a tweet was created in and add custom lexicons for them. Since this exceeds the scope of this research, we generated the sentiment scores for tweets in English only.

In March of 2021, we queried the Twitter API with each of the collected tweet’s ID to check the status of the tweet and its creator. After applying all filtration steps (non-English language exclusion, creation date exclusion, and duplicates removal), we ended up with 7,609,756 tweets, spanning ten days, that we used for this study. We further queried the Twitter API to store user-relevant information. Donald Trump was mentioned almost twice as many times as Joe Biden (Fig. 1), a common theme observed throughout the election cycle. There was a small percentage of tweets that mentioned both candidates as well.

**Fig. 2:**
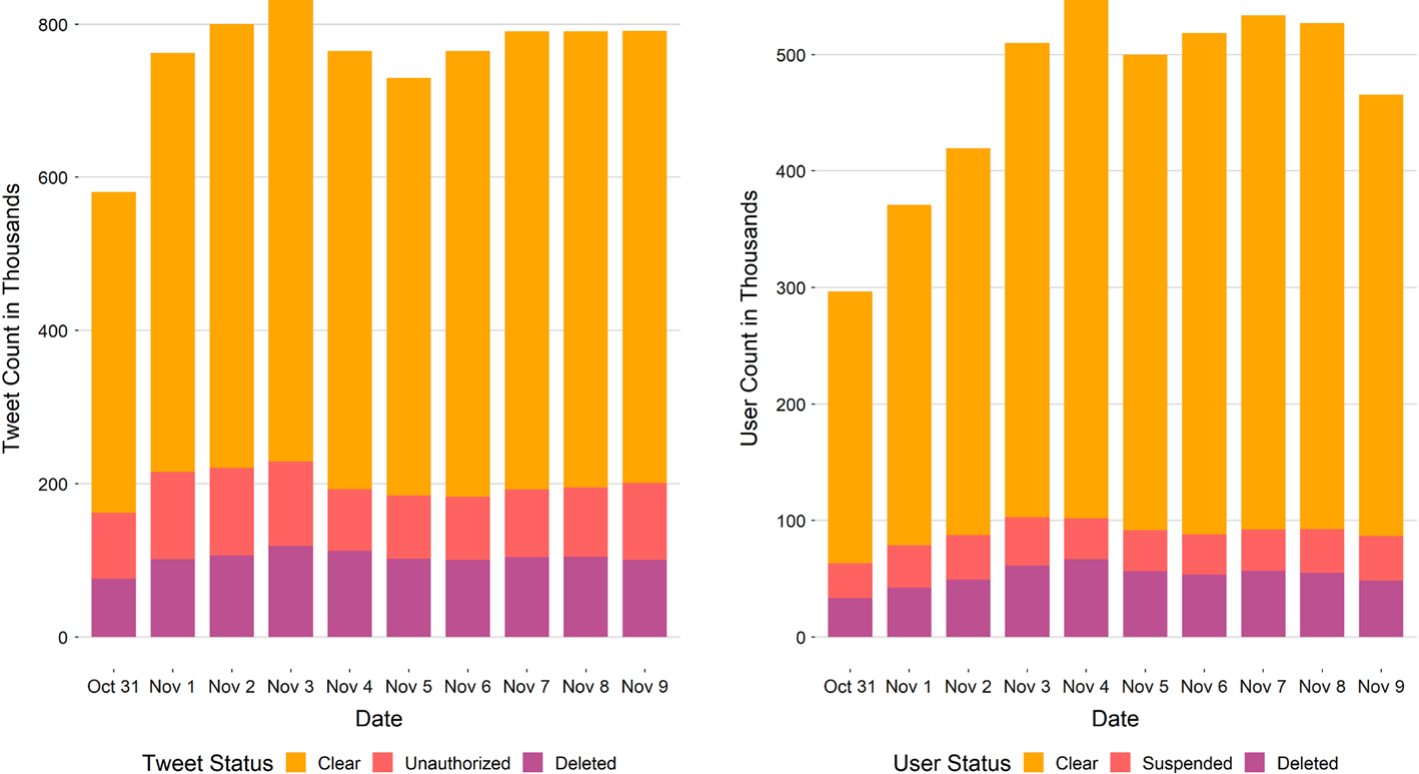
**a** Count of tweets per tweet status type per day. **b** Unique number of twitter users per user status type per day

### Methodology

Texts from social media platforms, like Twitter, tend to be noisy with non-text entities, which decreases the accuracy of the sentiment scores assigned to them. Excessive punctuation, emoji, and acronyms are usually ignored by popular sentiment model implementations. Studies, though, have emphasized the effect of slang, acronyms, and misspellings on the overall sentiment [9, 37, 38]. We, therefore, use the Valence Aware Dictionary and sEntiment Reasoner (VADER) sentiment analysis tool for our study, as it is specifically tuned for sentiments expressed in social media posts [39]. VADER incorporates negations, use of excessive punctuation, slang words, emojis, and acronyms, to accurately measure the sentiment of a document. As VADER automatically takes care of edge cases in text pre-processing and performs the removal of stop words and other steps required for Sentiment Analysis, the application of the tool is reasonably straightforward. We first remove URL links, numbers, and referenced Twitter user mentions (with @), using regular expressions. Then, VADER is applied to the data, which provides a compound score to determine the sentiment of each tweet. The sentiment score ranges from − 1 (very negative) to + 1 (very positive), and tweets with a score between − 0.05 and + 0.05 are deemed as having a neutral sentiment.

**Fig. 3:**
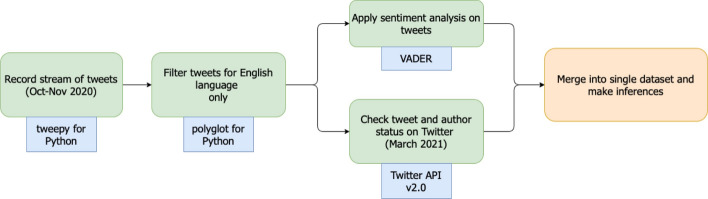
Flowchart of steps used in this study. The tool/package used for each step is added in the accompanying blue rectangle

Next, we create buckets of tweets based on the mentioned candidate, tweet status, and the user status. A tweet can have a deleted status: tweet that was later deleted by the creator, an unauthorized status: user who created the tweet either deleted their account or is currently suspended by Twitter, or a clear status: tweet is accessible. The user can also have a deleted status: account has been deleted by the user, a suspended status: account is currently suspended by Twitter for violations of any rules, and a clear status: account is accessible. Around 75% of all tweets in our dataset had a clear status (Fig. 2a). We observed a similar percentage for clear-status user accounts as well (Fig. 2b). While traffic of tweets mentioning the candidates was similar each day, the unique number of users contributing to that conversation increased daily until it plateaued on Election Day. A day saw, on average, 760,000 tweets and 469,000 unique users who engaged in candidate related conversations, across our study time period. Figure 3 shows the order of steps for techniques described in the data collection and methodology subsections.

**Fig. 4:**
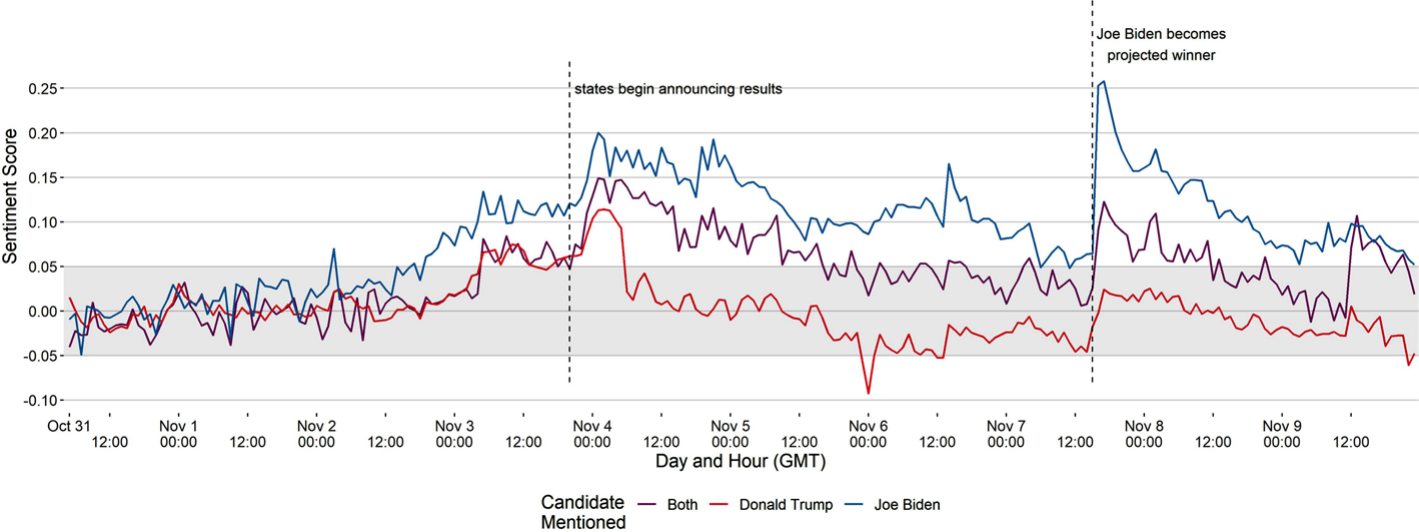
Timeline of sentiment scores of tweets that mentioned presidential candidates. Average sentiment scores are computed per hour for the duration of the study days. Timezone for date time is in GMT

## Results

We first present the average sentiment score recorded per hour for each candidate for the duration of the study (Fig. 4). Overall, Donald Trump had a sentiment score of 0.001, which falls under the neutral sentiment (– 0.05 to 0.05). On the other hand, Joe Biden had a score of 0.097, which falls under the positive sentiment. Tweets that mentioned both candidates had an average score of 0.041. We note that each candidate received a similar sentiment leading up to the elections, even though Donald Trump was talked about significantly more. Beginning with Election Day, sentiment around Joe Biden became increasingly positive. At the same time, Donald Trump’s remained within the neutral sentiment threshold, represented as the greyed-out zone on the plot. The sentiment of tweets that mentioned both candidates followed similar patterns. We manually analyzed subsets of tweets to verify the sentiment score assigned to them by VADER. Except for a few cases where sarcasm was not detected, we saw VADER making accurate sentiment classifications.

We further highlight two key events that happened during the ten days. First, initial election results from several states started pouring in around midnight (GMT) of November 4th , which saw a positive increase in the sentiment of each candidate. However, it continued to remain positive for Joe Biden. Early results from competitive states were encouraging for him, leading to a positive sentiment on Twitter [40]. The second key event occurred when Joe Biden was announced the projected winner of the elections on November 7th. The vote counting process would continue for several more weeks, but projections on November 7th showed that Joe Biden would win enough state delegates to become the president-elect [10, 40]. Consequently Joe Biden’s sentiment on Twitter became more positive, which would eventually return to near neutral in the days to come, as talks of a fraudulent election started to emerge on social media platforms [41, 42], shedding negative light on the eventual Democratic winner.

**Fig. 5:**
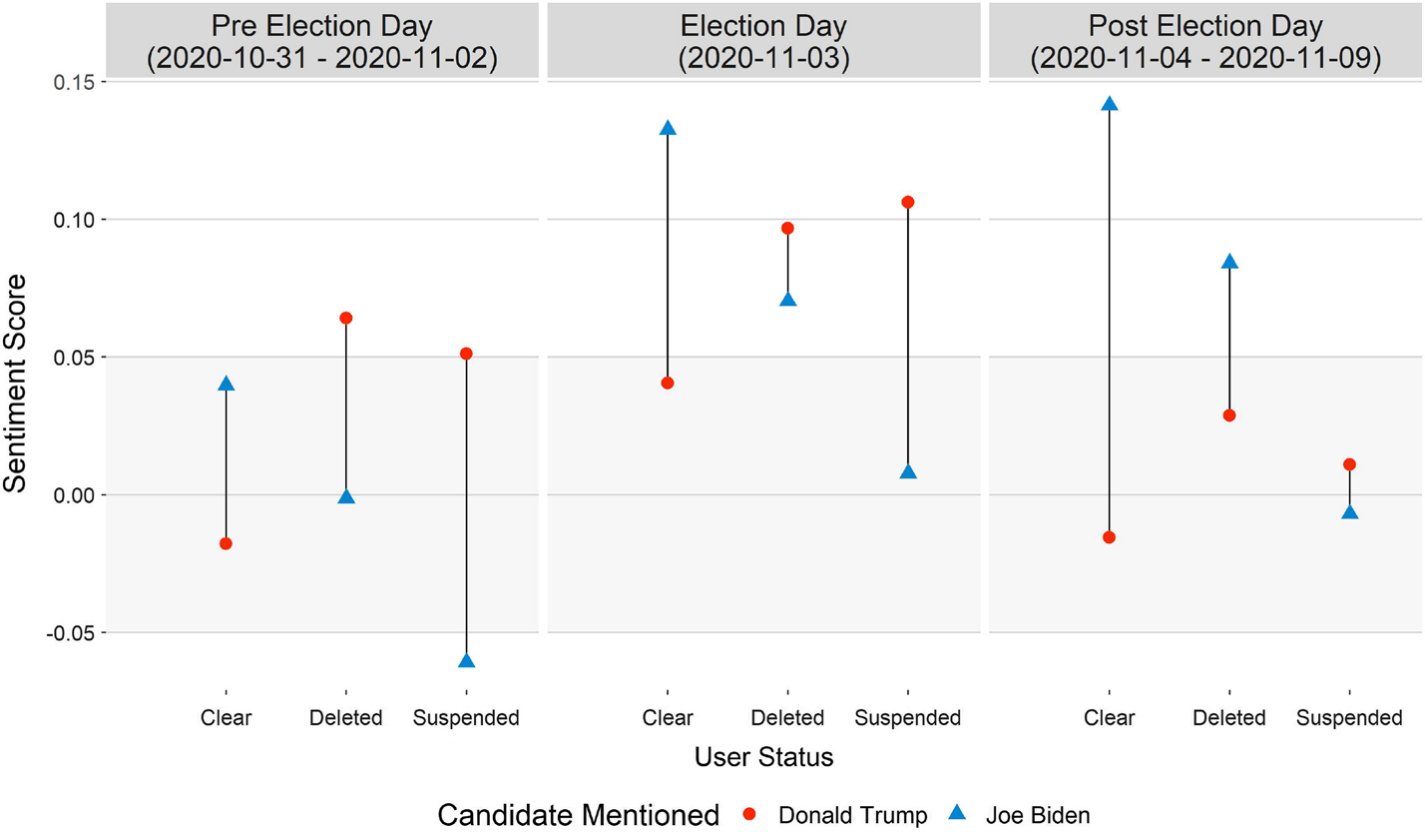
Average sentiment scores of tweets that mentioned either candidate grouped by user status type, before, during, and after Election Day. For each user status type, a straight line is drawn to highlight the difference between the sentiment scores for each candidate. They gray shaded region in the plots represents the neutral sentiment area. Tweets that mentioned both candidates are excluded

Looking deeper into the sentiment across different user status types, we plot the average sentiment score of tweets that mention either candidate, across three key date ranges: three days leading up to Election Day, the Election Day itself, and the six days after it (Fig. 5). For each user status type, across the three date ranges, we then map the sentiment scores for each candidate and draw a straight line to highlight the difference in the scores. The gray shaded area represents the neutral sentiment region. This visualization approach makes it easy to view how sentiment scores changed between user groups across different date ranges in the election cycle. Users with a clear status tweeted more positively about Joe Biden. Interestingly, users who were later suspended from Twitter, consistently held a negative sentiment towards the Democrat. In fact, tweets posted by suspended users during the first date range had an overall negative sentiment towards Joe Biden and a positive average sentiment towards the incumbent. While it is difficult to predict why these accounts were suspended, it is clear that they held a particular sentiment towards the Democratic challenger, which was not seen among clear status users. For the average sentiment shown from accounts that no longer exist on Twitter, we see that users with the deleted status held a more favorable sentiment towards Donald Trump. Notably, these 860,000 users tweeted positively about both candidates on Election Day. Contrary to our initial hypothesis, the deleted accounts were more positive about Joe Biden after November 3rd. Overall, however, only the clear status type users held a more positive sentiment towards Joe Biden over Donald Trump across all ten days.

**Fig. 6:**
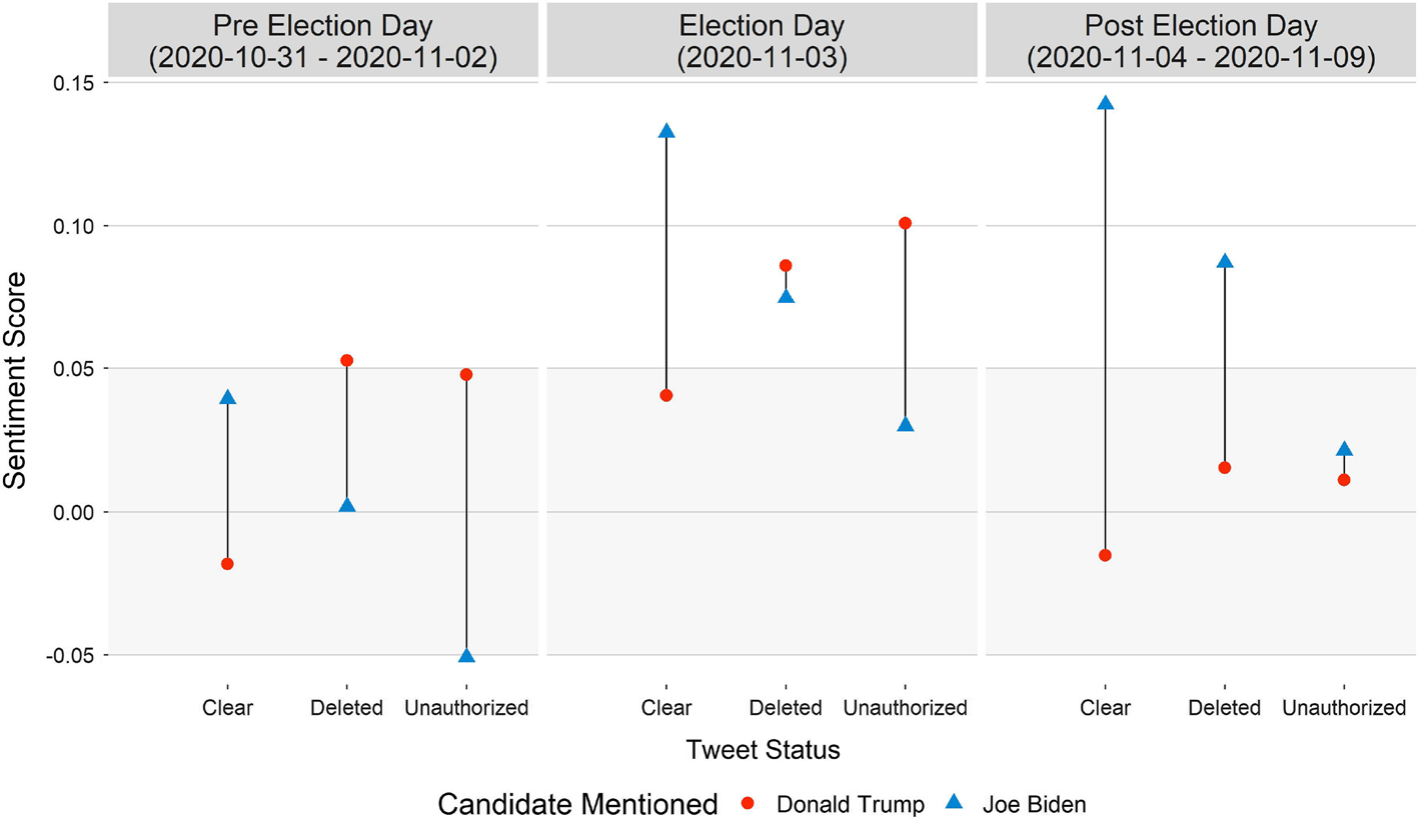
Average sentiment scores of tweets that mentioned either candidate grouped by tweet status type, before, during, and after Election Day. For each tweet status type, a straight line is drawn to highlight the difference between the sentiment scores for each candidate. They gray shaded region in the plots represents the neutral sentiment area. Tweets that mentioned both candidates are excluded

Next, we perform a similar analysis for different tweet status types (Fig. 6). First, we make an observation that the trends here are similar to those found for different user status types in terms of average sentiments and differences across date ranges. Tweets with the clear status had a positive sentiment towards Joe Biden, especially during and after Election Day. The unauthorized tweets posted by users who are suspended or no longer have an account held Donald Trump to a more favorable sentiment. Deleted tweets had a comparatively positive sentiment towards the president leading to Election Day, but after it, they had a much more positive sentiment when mentioning Joe Biden. This is as if there are two different groups of users, where the former may have removed their posts after their candidate was seemingly losing, and the latter may have removed their posts after allegations of voter fraud came to light.

We also discovered roughly sixty thousand Twitter users who had their tweets protected: only the accounts’ followers can view tweets posted by these users. Among such users, tweets that mentioned Joe Biden had a sentiment score of 0.145 (positive) versus Donald Trump’s sentiment score of 0.010 (neutral). Users with protected accounts can choose who follows them and can tweet knowing that only those they choose are able to view or reply to their tweets, simulating a closed group rather than an open forum. The sentiment score for Joe Biden from these users’ tweets exceeds the overall average sentiment for the Democrat (0.090), leading us to hypothesize that these users felt more comfortable having a political discussion about Joe Biden without potentially engaging in a heated argument with supporters of Donald Trump. This scenario is also studied by Gupta et al. [43] in their paper, analyzing political echo chambers on Twitter.

Lastly, we analyze the sentiment disparity for the two candidates over how old the Twitter accounts tweeting about them were. We subset accounts with a clear status only, as we could not extract further information for deleted and suspended accounts. We create five exclusive groups of Twitter accounts, with October 31st, 2020 being the starting date of our data collection process, and present sentiment scores across them (Table 1). We find that the older the account is, the more positive sentiment it holds for Joe Biden. The opposite is true for Donald Trump though his sentiment remained neutral overall. We also uncovered 27,000 accounts that were created after we began recording the tweets. Among these users, sentiment for Donald Trump was neutral (0.036), and that for Joe Biden was comparatively very positive (0.128). It should be noted, however, that accounts created after Joe Biden was announced the projected winner, on November 7th, posted tweets about him with a sentiment score of 0.213, the highest magnitude across any user or tweet group, which skews his favorability among new accounts.

**Table 1:** Sentiment score of tweets per presidential candidate by groups of accounts based on when they were created

Account Created	Number of Accounts	Biden’s Sentiment Score	Trump’s Sentiment Score
Within 10 days	15,008	0.041	0.005
Within 30 days	30,047	0.081	0.015
Within 1 year	298,010	0.097	− 0.003
Within 5 years	591,881	0.112	− 0.007
Older than 5 years	1,230,902	0.129	− 0.015

All groups are exclusive and are created with respect to October 31st, 2020 (the first day of our tweet collection process)

## Discussion

While Twitter automatically removes bots from the platform, if suspended, they get incorporated into the suspended user status type for further analysis. We only found ten accounts that posted, on average, 100 or more tweets per day. This still did not match the high activity rates of bots, studied by Kollanyi, Howard, and Woolley, in their paper on automation on Twitter during the 2016 U.S. Presidential Elections [44]. We also cannot determine when a tweet was removed or when a user was banned from Twitter or deleted their account entirely. This information would be valuable in further estimating why this action was taken and if it is relevant to the tweets the users posted during the election cycle.

Given just ten days of study period, we can still highlight key areas where sentiment shifted for a candidate with time. While causes behind a tweet deletion have been studied that could be replicated for this article [22], the disparity of sentiment between a different tweet and user status types for each presidential candidate is of significant importance. Joe Biden is a seasoned politician who was the Vice President for the very popular President Barack Obama. On the other hand, Donald Trump has remained a controversial figure throughout his presidency, garnering equal support and opposition in the US, a fact that is reflected in his overall neutral sentiment in tweets that mention him. Suspended and deleted Twitter accounts posted tweets with an overall positive sentiment when mentioning Donald Trump, but only until Election Day. Tweets and users with a clear status highly favored Joe Biden over Donald Trump throughout the study period. News reports citing a landslide victory for the challenger buoyed the sentiment for Joe Biden. However, it was not until he was announced as the projected winner by news outlets did the tweets that mentioned him became vehemently positive.

If we had conducted a sentiment analysis on clear tweets only, which account for 80-85% of daily traffic in our study, we would have concluded that Joe Biden had a clear favorability over Donald Trump, across all users. We would not have been able to ascertain a group of users who deleted their tweets later but held a more positive sentiment towards Donald Trump. Their inclusion in the study is a true reflection of what a social media platform like Twitter is; not all users are like-minded. If posts and accounts are removed at a later date that does not mean that those posts did not impact the conversations happening in real time. The true, raw sentiment of users during the event cannot be captured without these posts.

While sentiment analysis of tweets and understanding the causes and frequency of removed/suspended Twitter accounts and tweets have previously been studied, our approach is a novel one. We combine the two fields of research to understand the sentiment across such groups of accounts and tweets. Our methodology and data collection process make this study scalable to understand the sentiment shifts of entities in other news-worthy areas as well. We hope that future studies that focus on sentiment analysis of tweets also consider inclusion of deleted, removed, and suspended tweets and user accounts.

## Conclusion

Through the use of sentiment analysis and groupings of different users and their tweets, we provide useful insight into how political conversations, at a time of major political events, are conducted wholly on a social platform like Twitter. This is the first study of its kind; in which we show how the sentiment surrounding each candidate changed during ten key days of the 2020 U.S. Election cycle. We also demonstrated how users decided to post opinions with the knowledge that Twitter can suspend them or they can themselves remove their opinions at a later date. Not only were we able to show that a political tweet that was sent out before Election Day and was later deleted is more likely to be favorable towards Donald Trump, but we were also able to ascertain an inverse relation between the pessimism towards Joe Biden and how old a Twitter account is. Retrieval of inaccessible tweets is impossible without prior storage, and future studies should make an effort to include such tweets in their analyses to underscore the true sentiments felt at the time around major events.

## Data Availability

The datasets used and/or analysed during the current study are available from the corresponding author on reasonable request.

## Abbreviations

VADER: Valence Aware Dictionary and sEntiment Reasoner
